# Acute exogenous lipoid pneumonia leading to severe ARDS: a case report

**DOI:** 10.1186/s12890-025-03780-0

**Published:** 2025-07-02

**Authors:** Youdi Ye, Hui Cai, Chunfeng Dai, Qin Hu, Qiaoqiao Cao, Jing Bi, Yuanlin Song, Jinjun Jiang, Shujing Chen

**Affiliations:** 1 Department of Pulmonary and Critical Care Medicine, Huangshan People’s Hospital, Huangshan, Anhui China; 2 Department of Pulmonary and Critical Care Medicine, Zhongshan Hospital, Fudan University, Shanghai, China; 3 Department of Cardiology, Zhongshan Hospital, Fudan University, Shanghai, China; 4 Department of Pathology, Zhongshan Hospital, Fudan University, Shanghai, China; 5 Department of Pulmonary and Critical Care Medicine, Nantong Haimen District People’s Hospital, Nantong, Jiangsu China

**Keywords:** Exogenous lipoid pneumonia, Acute respiratory distress syndrome, Bronchoalveolar lavage, Mechanical ventilation, Prone position ventilation

## Abstract

**Background:**

Exogenous lipoid pneumonia, a form of pneumonia caused by the aspiration of lipid substances, is often associated with the use of mineral oil. Historically, cases have predominantly been reported in young children, elderly, or individuals with compromised physical strength or neurological disorders, with the majority presenting as mild or chronic conditions. Upon cessation of exposure to lipids, symptoms typically showed improvement.

**Case presentation:**

We report a case of a previously healthy middle-aged man who developed respiratory failure and severe acute respiratory distress syndrome (ARDS) following the accidental aspiration of sewing machine oil, and a chest computed tomography (CT) scan revealed consolidations in both lungs, with local attenuation visible in the mediastinal window. Lipid vacuoles were observed in the bronchoalveolar lavage fluid, and Oil Red O staining was positive, confirming the diagnosis. In terms of treatment, invasive mechanical ventilation was provided, along with intermittent prone positioning ventilation, segmented alveolar lavage, and systemic corticosteroids as part of a comprehensive treatment approach. The patient’s oxygenation gradually improved, leading to stabilization, and follow-up chest CT three months later showed resolution of the lung lesions.

**Conclusion:**

The diagnosis of exogenous lipoid pneumonia is based on a history of lipid exposure, chest imaging, and the presence of lipid-laden macrophages in bronchoalveolar lavage fluid. However, there is currently no established treatment protocol. For critically ill patients, life support measures are crucial during the early stages or peak of the disease.

**Clinical trial number:**

Not applicable.

## Background

Exogenous lipoid pneumonia (ELP) was initially described by Laughlen in 1925 [1]. It is uncommon, although it is difficult to determine the precise clinical incidence, autopsy series have reported a frequency of only 1.0–2.5% [2], with higher rates observed among patients with neurological disorders and gastroesophageal reflux disease [3, 4]. Occupational exposure such as chronic inhalation of cutting fluid mists and oily vapors in industrial settings, as well as activities like fire-eating performances, have also been reported to be causing ELP [5, 6]. The diagnosis of ELP is based on a history of exposure to oils, characteristic radiological findings, and the presence of lipid-laden macrophages in bronchoalveolar lavage (BAL) fluid analysis [7]. However, to date, no standard treatment regimen has been established. Therefore, we present a successfully treated case of severe ELP aiming to contribute to the development of such regimen.

## Case presentation

**Fig. 1:**
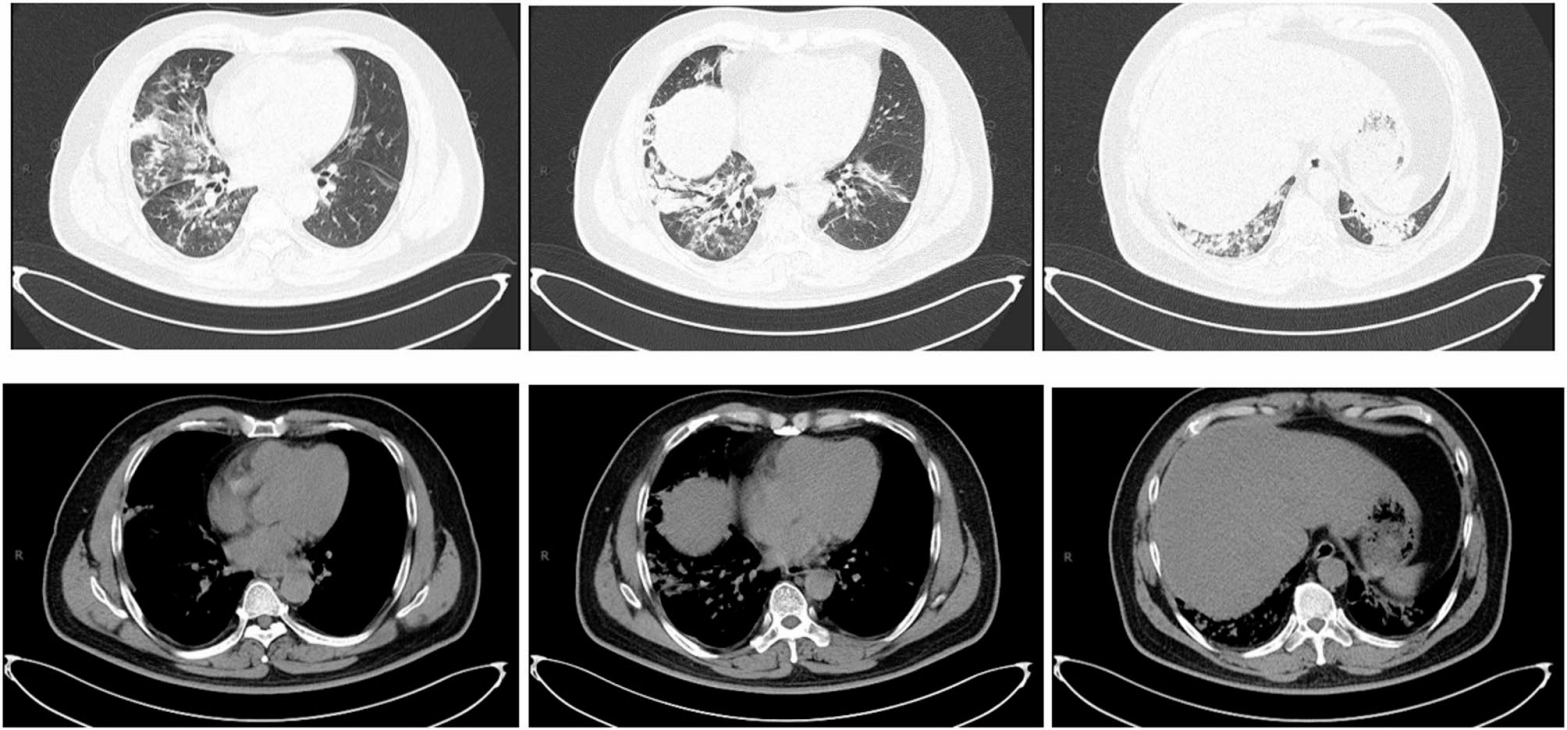
Chest CT scan showed multifocal consolidative opacities on two days before admission to Zhongshan Hospital

A previously healthy 50-year-old man was admitted to the local hospital due to symptoms of cough and fever. One week prior to admission, while driving, he accidentally drank from a bottle containing sewing machine oil, which lead to have a chocking cough. The following day, he developed a high fever up to 40 °C, accompanied by severe dry cough, and was eventually hospitalized. Initial blood tests revealed a white blood cell (WBC) count of 11.6 × 10^9/L and a C-reactive protein (CRP) level of 161 mg/L. Arterial blood gas (ABG) analysis showed a pH of 7.51, PCO2 of 25 mmHg, and a PaO2 of 57 mmHg on 2 L/min of oxygen, resulting in a PaO_2_/FiO_2_ (P/F) ratio of 196. A chest CT scan revealed bilateral pneumonia (Fig. 1). Despite a week of treatment with biapenem and moxifloxacin, his condition deteriorated, with worsening respiratory distress. The patient was then referred to Zhongshan Hospital affiliated with Fudan University.

**Fig. 2:**
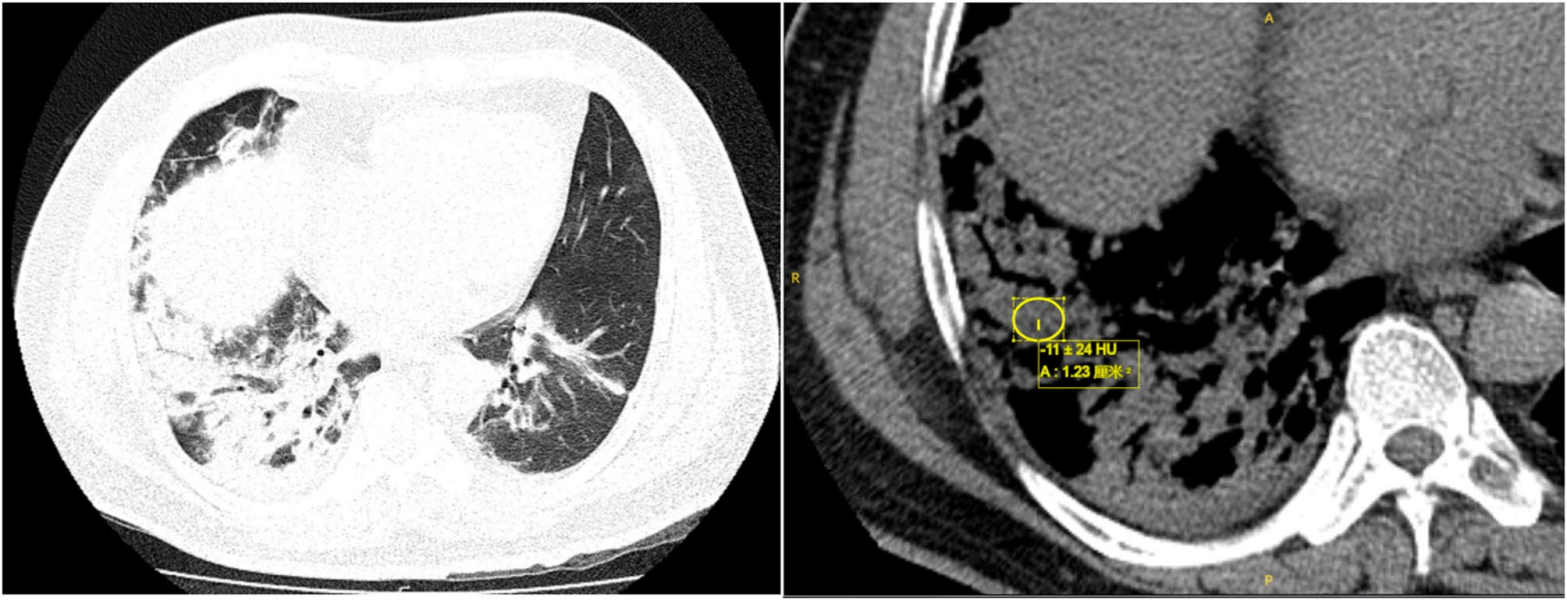
Chest CT showed multifocal consolidative opacities with areas of low attenuation on the first day of admission to Zhongshan Hospital

On admission, the patient presented to the emergency department with a temperature of 37.4 °C, heart rate of 120 bpm, respiratory rate of 25 breaths per minute, and oxygen saturation of 88% on 6 L/min nasal cannula oxygen. Upon physical examination the patient was conscious with a blood pressure was 131/82 mmHg. Lung auscultation revealed clear breath sounds on the left and coarse on the right, without significant rale. Labs showed a WBC count of 13.54 × 10^9/L and a CRP > 90 mg/L; liver and kidney functions, electrolytes, blood sugar, cardiac markers, and coagulation profiles were essentially normal. ABG analysis revealed a PaO2 of 62.47mmHg and a P/F ratio of 152. And a follow-up chest CT scan (Fig. 2) showed progression of exudate and consolidation in both lungs compared to previous findings, with more pronounced changes on the right and with local low-density areas in the consolidated regions, CT values − 32 to 13 HU. The patient was admitted to the Emergency Intensive Care Unit (EICU), treated with high-flow nasal oxygen therapy, moxifloxacin for infection, and methylprednisolone 40 mg qd for inflammation.

**Fig. 3:**
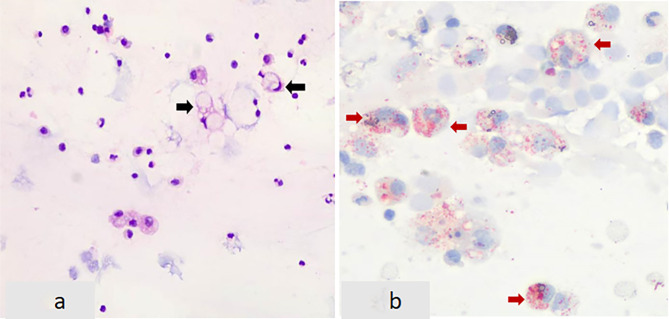
**a** The alveolar lavage fluid smear shows lipid vacuoles within tissue cells (indicated by black arrows). **b** Oil Red O staining is positive under high magnification (indicated by red arrows)

**Fig. 4:**
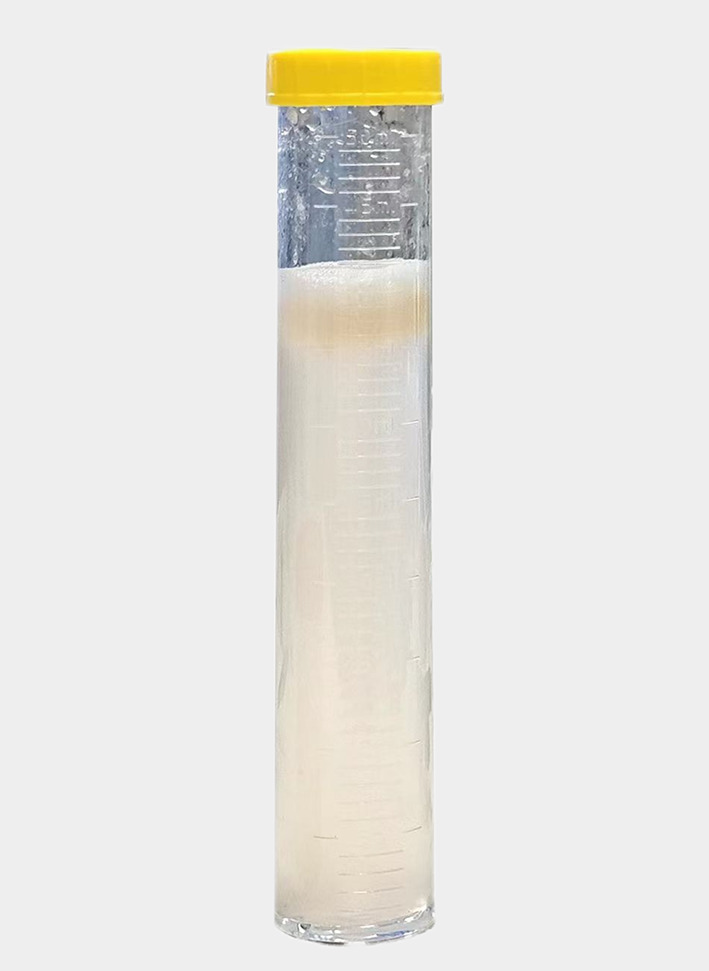
The alveolar lavage fluid is turbid and layered, with a milky yellow oily liquid visible on the surface after settling

**Fig. 5:**
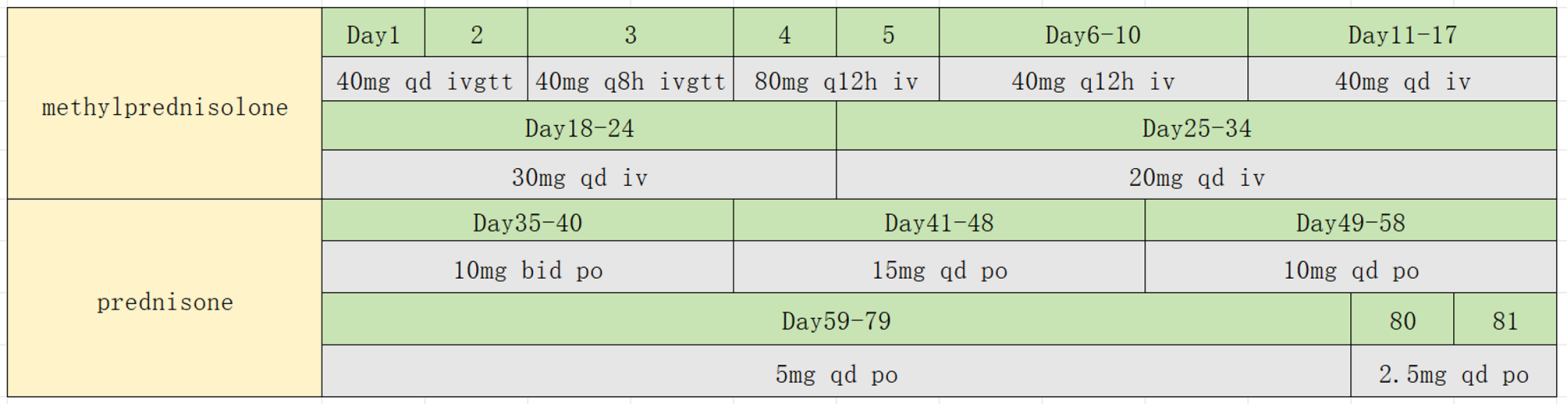
The duration and dosage of corticosteroid used for the patient

**Fig. 6:**
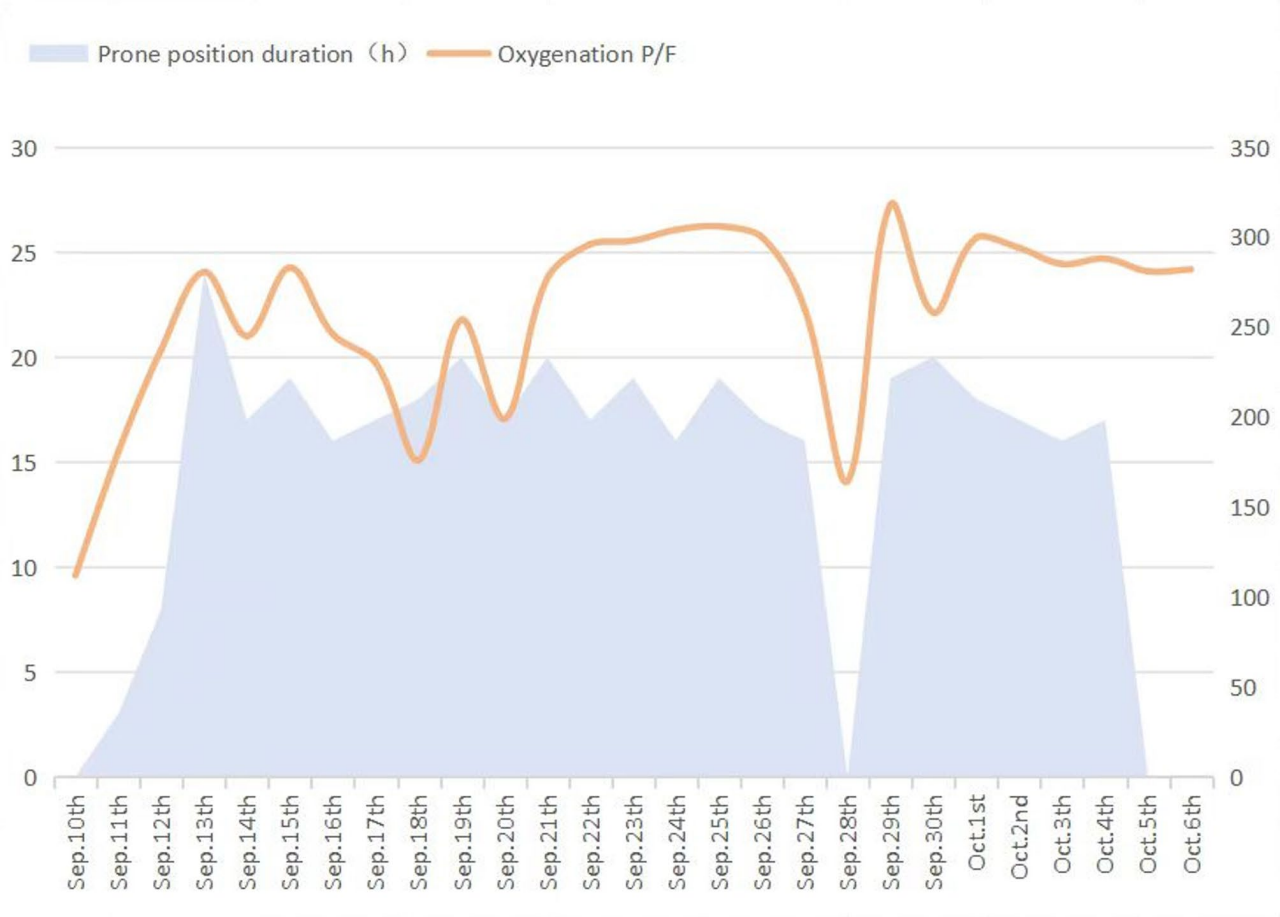
Oxygenation varies with duration of prone positioning

**Fig. 7:**
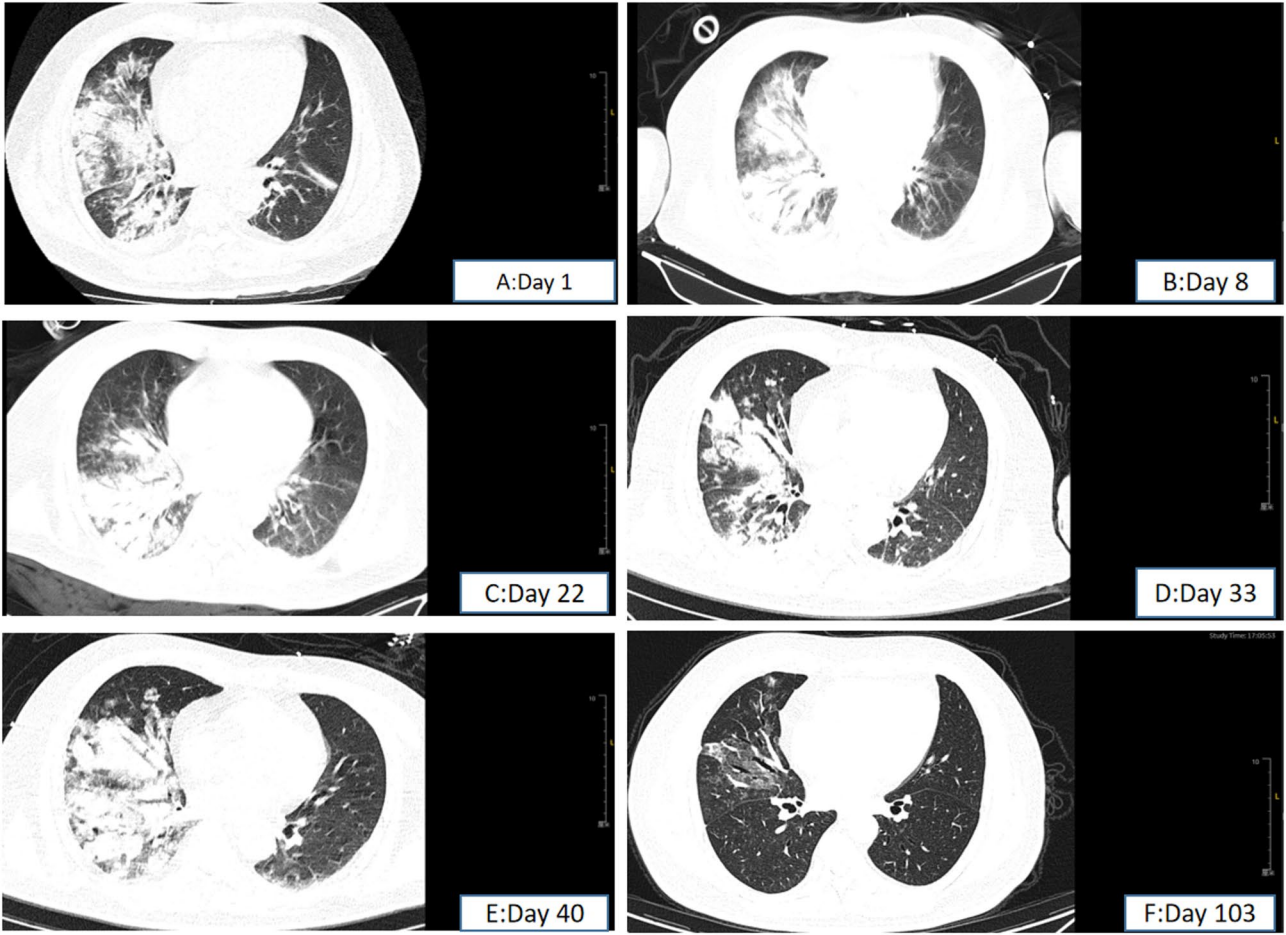
Changes in chest CT during the treatment. The day 1 is September 6th, the day of admission to Zhongshan Hospital

However, he showed no improvement and was therefore transferred to RICU on the 4th day since the initial hospitalization at Zhongshan Hospital. Initial ABG under high-flow oxygen showed a P/F ratio 112, and a ROX index of 5.4. Prompt treatment involved continuation of high-flow oxygen, administration of meropenem for infection, and methylprednisolone (80 mg q12h IV) for inflammation. On the 5th day, prone positioning was attempted, but it could not be tolerated due to patient’s chest tightness and dyspnea. Within 12 h, his P/F ratio dropped to 93, and the ROX index decreased to 4.0. As a result, intubation, lung protective ventilation and prone positioning were initiated. Bronchoscopy and alveolar lavage post-intubation showed no secretions. Lavage fluid cytology: macrophages 86%, lymphocytes 12%, neutrophils 2%, eosinophils 0.0%. On the 8th day, sputum culture revealed multidrug-resistant Klebsiella pneumoniae sensitive to colistin, tigecycline, and ceftazidime-avibactam. Chylous test was positive, and smear and special staining showed ciliated and tissue cells with lipid vacuoles, positive for Oil Red O (Fig. 3). Clinical history, imaging and pathology results confirmed the diagnosis of ELP. Hence, the patient was placed on continuous invasive mechanical ventilation. Initially, ventilation was performed in the prone position for 44 h. Then, once a P/F ratio of 245.25 was achieved, the patient was transitioned to a regimen of daily prone ventilation sessions lasting more than 16 h. Serial bronchoscopic alveolar lavages were conducted, with turbid fluid retrieved from different lung segments on each occasion (Fig. 4). Positive chylous tests on days 14, 18, 24, and 34, along with lipid vacuoles observed in lavage cells on days 7, 14, and 38, indicated the ongoing presence of ELP. Treatment included ceftazidime-avibactam and colistin for multidrug-resistant *Klebsiella pneumoniae*, along with corticosteroids (Fig. 5), sivelestat sodium hydrate, and immunoglobulins. As a consequence, oxygenation improved significantly (Fig. 6). Regular blood tests for inflammatory markers and chest imaging showed no significant signs of secondary infection. A tracheostomy on day 33 facilitated weaning from mechanical ventilation and initiation of high-flow oxygen therapy and respiratory rehabilitation. The patient was discharged on day 41 and continued outpatient rehabilitation. Full weaning was achieved on day 67 and the tracheostomy tube was removed on day 78. On day 103, a follow-up CT showed significant resolution of lung lesions (Fig. 7), the patient had no longer any symptoms and was active, with an oxygen saturation of 98% on room air.

## Discussion

This is a case of exogenous lipoid pneumonia with a definitive history of sewing machine oil aspiration. The condition progressed rapidly, with an oxygenation index below 100mmHg, indicating severe ARDS. Timely intubation and implementation of lung-protective mechanical ventilation, combined with prone positioning, improved the clinical outcome.

Lipoid pneumonia is an uncommon lung disease characterized by the presence of intrapulmonary lipids and lipid-laden macrophages under the microscope. It can be categorized into two types based on the source of lipids in the airways: endogenous and exogenous [8]. Endogenous lipoid pneumonia, involves lipids produced by the lung tissue itself. It is commonly associated with conditions such as pulmonary alveolar proteinosis, connective tissue diseases, and sclerosing cholangitis [9]. Exogenous lipoid pneumonia (ELP) is caused by the inhalation of lipids derived from animal, plant, or mineral sources. Most cases of ELP occur due to mineral oil, which can suppress the cough reflex and ciliary movement, thereby facilitating aspiration [5]. The mineral oils commonly encountered in adult settings are the ones used in constipation relief medication and in nasal drops for the treatment of rhinitis [3, 10]. In addition, ELP can be further classified into acute and chronic forms. Acute ELP is uncommon and usually results from aspiration of a large quantity of a petroleum-based product. Chronic ELP usually arises from repeated episodes of aspiration or the inhalation of fatty substances over an extended period [3].While the exact mechanism of lipid-induced lung damage is not fully understood, it is believed that inhaled lipids are emulsified and phagocytosed by alveolar macrophages. After the macrophages disintegrate, lipids are then liberated into the alveoli, triggering chronic inflammation and ultimately leading to fibrosis [11].

The patient in this report aspirated a sip of sewing machine oil and developed high fever and dry cough the next day. A chest CT showed bilateral lung infiltrates, and blood gas analysis indicated Type I respiratory failure. The onset was sudden and the condition progressed rapidly. Research on the relationship between the type and amount of inhaled mineral oil and the severity of lipoid pneumonia remains limited. Wan-ding Ye reported that high dose oil exposure can trigger excessive inflammation that can potentially cause acute respiratory failure or death [12].However, Sen Yang’s study found no significant correlation between the type of oil and laboratory findings or the prognosis of ELP [13].

ELP lacks specific clinical manifestations, with symptoms varying significantly among individuals, ranging from asymptomatic to severe, life-threatening conditions. In this case, the patient presented with high fever, dry cough, and progressive dyspnea, symptoms consistent with previous reports [5].

Acute ELP typically shows consolidation, ground-glass opacities, nodules, and crazy-paving patterns on lung CT, with consolidation often uneven and CT values ranging from − 30 to -150 HU, potentially increased by overlapping inflammation [14]. Lesions are usually peribronchovascular and gravity-dependent. In this case, low-density areas within right lower lobe consolidation had CT values of -32 to 13 HU, consistent with lipoid pneumonia. Imaging findings of acute ELP often partially or completely resolve over time after lipid exposure ceases, with shadows typically disappearing within two to eight months [2, 15]. In this patient, right middle and lower lobe consolidation showed no significant absorption after four weeks of treatment, but showed marked resolution after three months.

In ELP, bronchoalveolar lavage (BAL) fluid may appear white or turbid, with oily substances floating on the surface, as observed in this case. Midulla et al. reported that the most significant BAL findings include an increase in lipid-laden macrophages, a significant decrease in normal alveolar macrophages, a slight increase in eosinophils, and an increase in activated lymphocytes [16]. In this case, the BAL cell count showed normal, while multiple chylous tests were positive, and lipid vacuoles were observed in some tissue cells, with positive Oil Red O staining. Combined with the patient’s history of mineral oil aspiration and chest imaging changes, the diagnosis of lipoid pneumonia was confirmed.

Currently, treatment of lipoid pneumonia is not well studied, most published treatment experience is limited to case reports. The key measure is to eliminate exposure to the causative substances, emphasizing prevention, particularly in workplaces. Additional treatments include oxygen therapy, corticosteroid treatment, and mechanical removal of lipids from the lungs [9].

Previous reports have primarily documented mild or chronic cases of ELP without respiratory failure, and symptoms or chest CT manifestations often improved after halting the exposure or treated with corticosteroids [3, 4, 6, 7, 10, 16–18]. Hideki Yasui et al. reported a case of severe acute ELP with Type I respiratory failure, but the patient improved after a few days of corticosteroid treatment.

In our report, the patient presented with severe exogenous lipoid pneumonia, exhibiting respiratory failure upon admission, with chest imaging showing extensive consolidations in both lungs, predominantly in the right middle and lower lobes. ABG analysis revealed a P/F ratio of 93, meeting ARDS criteria after excluding cardiac causes [19]. Initially, the patient was guided to undergo awake prone positioning. Several studies have shown that awake prone positioning can improve oxygenation and ROX index in patients with respiratory failure, reducing intubation and mortality rates [20–22]. However, due to the patient’s abdominal obesity (BMI 29.1 kg/m²) and severe dyspnea, awake prone positioning could not be tolerated, and despite high-flow nasal oxygen therapy, his oxygenation progressively worsened, with a declining ROX index. Consequently, the patient was intubated and placed on lung-protective ventilation combined with prone positioning. Prone positioning can increase functional residual capacity, improve ventilation-perfusion ratio in gravity-dependent lung areas, enhance diaphragmatic movement, and facilitate sputum drainage [19]. After two hours of prone positioning, the patient’s ABG showed a P/F ratio increased to 237.4, indicating significant improvement in oxygenation. The patient’s first prone ventilation lasted an extended duration of 44 h. Research indicates that compared to patients receiving intermittent prone positioning, those with severe ARDS who undergo extended prone positioning have lower mortality risks at 30 and 90 days, with more pronounced benefits in severe cases [21]. After 44 h of prone ventilation, the patient’s P/F ratio rose to 245.25. Extended prone positioning allows for longer-lasting physiological benefits, including enhanced alveolar recruitment and improved ventilation-perfusion, while preventing lung injury caused by interruptions. As shown in Fig. 5, the P/F ratio significantly increased with prolonged prone positioning, and it decreased when prone positioning was stopped on day 21, demonstrating the clear benefits of prone ventilation.

Whole lung lavage (WLL), sometimes used for symptomatic pulmonary alveolar proteinosis, has also been reported as an effective treatment for ELP, especially in the pediatric population when combined with immunoglobin [13, 23]. This is reflected on CT scans findings, oxygen saturation levels, and lavage fluid cell composition. In this case, due to the need for invasive mechanical ventilation and prone positioning, bedside segmental bronchoalveolar lavage (sBAL) was performed. Shang et al. [24] noted in a systematic review that sBAL is safer and can be performed with a simpler procedure, while WLL has the potential to be more powerful and effective in clearing the aspirated substances and inflammatory factors. In one case report, WLL was initially planned for a 2-year-old infant but was replaced with sBAL because the patient could not maintain satisfactory oxygen saturation during the procedure [24]. Repeat BAL can play a crucial role in the management of ELP. It also helps in identifying any secondary infections that may complicate the clinical course. In our case, repeat BAL was instrumental in confirming the persistence of lipid-laden macrophages and ruling out secondary bacterial infections. However, the therapeutic role of BAL in acute cases has yet to be evaluated.

Differentiating between infectious pneumonia and exogenous lipoid pneumonia (ELP) can be challenging due to overlapping clinical and radiological features. While both conditions can present with cough, fever, and radiological evidence of consolidation, there are key differences that can aid in diagnosis. Infectious pneumonia often presents with purulent sputum and elevated inflammatory markers, whereas ELP typically shows lipid-laden macrophages on BAL fluid analysis. Radiologically, ELP may show characteristic findings such as the ‘crazy-paving’ pattern [15]. Microbiological studies, including cultures and molecular diagnostics, are essential in ruling out infectious causes. In our case, the absence of purulent sputum along with the presence of lipid-laden macrophages on BAL, supported the diagnosis of ELP. Otherwise, the patient had elevated white blood cell counts and CRP levels upon admission, and Klebsiella pneumoniae was detected in the bronchoalveolar lavage fluid, indicating a secondary bacterial infection in the context of lipoid pneumonia.

The use of systemic corticosteroids for treating lipoid pneumonia remains controversial, and is primarily reserved for cases of severe lung damage and persistent disease [25].There is no consensus on the dosage and duration of corticosteroid treatment for lipoid pneumonia. Hideki Yasui et al. [25] reported a severe aspiration pneumonia patient treated with 30 mg of prednisolone daily (60 kg man at a dose of 0.5 mg/kg), resulting in rapid fever relief and improved chest CT imaging after two months of steroid therapy. In contrast, Sen Yang et al. reported a retrospective study of 17 pediatric aspiration pneumonia cases, with a median corticosteroid dose of 1-2 mg/kg/day of oral prednisolone [13]. Peter V. Dicpinigaitis et al. reported a 28-year-old previously healthy man with vapingassociated acute respiratory failure due to acute lipoid pneumonia, empiric intravenous (IV) steroid therapy was begun with methylprednisolone 40 mg every 6 h. By hospital day 9 the patient demonstrated adequate oxygen saturation while breathing room air, and he was discharged home on hospital day 11 [26]. Muneyoshi Kuroyama [27] et al. reported the patient received steroid pulse therapy with methylprednisolone (1 g) for 3 days, followed by prednisolone (20 mg), resulting in a mild improvement. In our case, the patient presented with acute onset, severe symptoms, poor oxygenation, and rapid progression of pulmonary lesions, which led the EICU physicians to increase the dose of methylprednisolone (from 40 mg/day to 120 mg/day). After transfer to the RICU, the dose of methylprednisolone was temporarily increased to 160 mg/day for only 2 days before being rapidly tapered. This decision was primarily based on the confirmed diagnosis of lipoid pneumonia via bronchoalveolar lavage fluid analysis and the consideration of the risk of secondary infections. Once the methylprednisolone dose was reduced to below 20 mg/day, oral corticosteroids were sequentially administered. Typically, we opted for prednisone for oral treatment, which is related to the availability of the medication.

In the cited case reports above, patients have shown good therapeutic effects with two different types of corticosteroids [13, 25–27]. In our case, we initially chose 160 mg of intravenous methylprednisolone daily for 2 days, the patient’s oxygenation improved, but chest imaging showed slow resolution, likely due to elevated Krebs Van den Lungen-6(KL-6) levels. The patient’s KL-6 levels were 466U/ml on day 11 and increased to 1529U/ml on day 38. KL-6 is a glycoprotein widely present on the membranes of type II alveolar epithelial cells. When affected by fibrosis, it is shed from the alveolar epithelial cells and can be detected in the blood or alveolar space. KL-6 exacerbates lung damage, by promoting collagen production, inducing fibroblasts to differentiate into myofibroblasts, and producing more extracellular matrix, leading to deposition and remodeling, affecting lung ventilation. All these factors collectively accelerate the progression of pulmonary interstitial fibrosis [28].Corticosteroid may prevent fibrosis and lung capacity loss in acute severe cases. On day 35, the patient began taking oral prednisolone, a chest CT scan on day 103 showed significant improvement, indicating that the radiological resolution of lipoid pneumonia is a gradual process.

Current research is inconclusive regarding the duration of corticosteroid therapy that would provide tangible clinical benefits to patients. In previously reported cases, the duration of corticosteroid therapy ranged from 6 months to 1 year [3, 27, 29]. In our report, from a radiological perspective, organizing pneumonia was initially suspected, characterized by consolidation along the bronchovascular bundles. At discharge on Day 41, the corticosteroid dose had been tapered to 15 mg of prednisone once daily orally. The subsequent plan at the rehabilitation hospital was to reduce the dose by 5 mg every 7 to 10 days. During the course of taking one tablet per day, the patient felt well and forgot to discontinue the medication. However, it is likely due to the prolonged use of low-dose corticosteroids (which was sufficient) that on follow-up at Day 103, the patient’s pulmonary lesions were almost completely absorbed. Therefore, we speculate that low-dose corticosteroids may promote the absorption of lipoid pneumonia. This is also a point for readers to consider in treatment, and we hope that more cases can be shared and summarized.

Balancing the role of corticosteroids in suppressing inflammatory responses and increasing the risk of secondary infections is an art. We dynamically monitored inflammatory markers in the blood, radiological findings, and the volume and characteristics of lower respiratory tract secretions to achieve the best therapeutic effect with minimal side effects (including metabolic management). Regular blood tests for inflammatory markers and chest imaging showed no significant signs of secondary infection.

Lipoid pneumonia typically does not require surgical treatment, as it is generally indolent and may resolve spontaneously. However, in a case reported by Gondouin, a patient underwent lobectomy to treat recurrent infections in the region affected by lipoid pneumonia [5].

Chronic exogenous lipoid pneumonia is more common and results from the repeated aspiration of mineral oils/fatty substances of animal origin [5]. It usually presents with dyspnea and/or cough. Physical examination findings and blood investigation results are usually normal. Upon the research of B.F. Samhouri [30], chronic exogenous lipoid pneumonia often affected older individuals, especially those with comorbidities that increase the risk of aspiration, gastric regurgitation, or both. The classic feature of fatty attenuation on chest CT scan is present in less than one-half of patients.

And after discontinuation of the causative substance, only a small percentage of the patients improved clinically (25%) or radiologically (33%).

## Conclusions

The diagnosis of exogenous lipoid pneumonia is based on a history of lipid exposure, chest imaging, and the presence of lipid-laden macrophages in bronchoalveolar lavage fluid. However, there is currently no established treatment protocol. For critically ill patients, life support measures are crucial during the early stages or peak of the disease. We reported a case of a critically severe patient with a clear diagnosis of exogenous lipoid pneumonia. After aggressive treatment with lung-protective ventilation combined with prone positioning, multiple segmental BALs, and corticosteroids, the patient’s condition improved. Follow-up revealed significant improvement on chest imaging.

## Data Availability

All data generated or analyzed during this study are included in this article.

## Abbreviations

ARDS: Acute respiratory distress syndrome
ELP: Exogenous lipoid pneumonia
CT: Computed tomography
BAL: Bronchoalveolar lavage
WBC: White blood cell
CRP: C-reactive protein
ABG: Arterial blood gas
EICU: Emergency Intensive Care Unit
IV: Intravenous injection
ROX: Ratio of SpO2/FiO2 to respiratory rate
WLL: Whole lung lavage
KL-6: Krebs Van den Lungen-6
P/F: PaO_2_/FiO_2_
_sBAL_: Segmental bronchoalveolar lavage
